# Unbiased, comprehensive analysis of Japanese health checkup data reveals a protective effect of light to moderate alcohol consumption on lung function

**DOI:** 10.1038/s41598-021-95515-4

**Published:** 2021-08-05

**Authors:** Kanako Makino, Ryoko Shimizu-Hirota, Norio Goda, Masahiro Hashimoto, Ichiro Kawada, Kazuhiro Kashiwagi, Yasushi Hirota, Hiroshi Itoh, Masahiro Jinzaki, Yasushi Iwao, Minoru Ko, Shigeru Ko, Hiromasa Takaishi

**Affiliations:** 1 Center for Preventive Medicine, School of Medicine, Keio University, Tokyo, Japan; 2 Department of Systems Medicine, School of Medicine, Keio University, Tokyo, Japan; 3 Department of Radiology, School of Medicine, Keio University, Tokyo, Japan; 4 Department of Pulmonary Medicine, School of Medicine, Keio University, Tokyo, Japan; 5 Department of Obstetrics and Gynecology, Graduate School of Medicine, The University of Tokyo, Tokyo, Japan; 6 Department of Nephrology, Endocrinology and Metabolism, School of Medicine, Keio University, Tokyo, Japan

**Keywords:** Epidemiology, Preclinical research

## Abstract

The overall effect of lifestyle habits, such as alcohol consumption, on general health remains controversial and it is important to clarify how such habits affect aging-related health impairments. To discover novel impacts of lifestyle on general health, we employed a mathematical approach to perform a comprehensive, unbiased, cross-sectional analysis of data from 6036 subjects who participated in a Japanese health checkup. Notably, we found that moderate alcohol consumption was positively correlated with lung function, muscle mass, and strength. Health checkup data were collected periodically from the same subjects. These people were light to moderate drinkers who had high health awareness and were basically free of major underlying diseases. We next analyzed 5 years of data from 1765 of these subjects. We found that higher baseline alcohol consumption, as well as increased alcohol intake over 5 years attenuated time-related deterioration of forced vital capacity without affecting total lung volume. This effect was independent of smoking. Our study suggests a possible protective effect of moderate amounts of alcohol on lung function, due to increased muscle mass/strength and forced vital capacity.

## Introduction

Lifestyle habits are critical for health. Along with genetics, unfavorable lifestyle habits such as smoking, heavy alcohol consumption, insufficient sleep, and infrequent exercise are major risk factors for cardiometabolic diseases^1^, which are increasing. However, health consequences of some lifestyle habits, such as alcohol consumption, remain controversial.

During the last decade, the Japan population has experienced an unprecedented increase in lifespan^2^, but age-related, physical, and functional deterioration have become nationwide problems, with increasing costs. Accordingly, there is great social demand to understand effects of lifestyle modifications on aging-related deterioration.

In April 2008, Japan initiated an annual health check-up, primarily for prevention of metabolic syndrome, for all people in Japan aged 40–74 years. This check-up includes standard medical examinations, and a questionnaire related to lifestyle. Data from these check-ups were collected not only from many people, but from the same people year after year, enabling large-scale longitudinal studies. Furthermore, most of the people who receive health check-ups are relatively healthy. This makes these health data ideal for temporal analyses of physiological age-related deterioration.

We performed an unbiased comprehensive analysis of data obtained from Japanese health checkups using a statistical approach to discover novel, unexpected impacts of lifestyle on health. We employed medical examination data and a questionnaire related to lifestyle. We used digital data from Keio University Hospital recorded over 5 years. Our comprehensive analysis of cross-sectional data using various statistical algorithms revealed an independent, positive correlation between total alcohol consumption and lung function. Subsequent analysis of longitudinal data showed that moderately higher baseline alcohol consumption, as well as an increase therein over 5 years, attenuated time-related deterioration in forced vital capacity (FVC) without affecting total lung volume. Our analyses highlight a previously unknown relationship between alcohol and health.

## Results

### Study population

**Figure 1:**
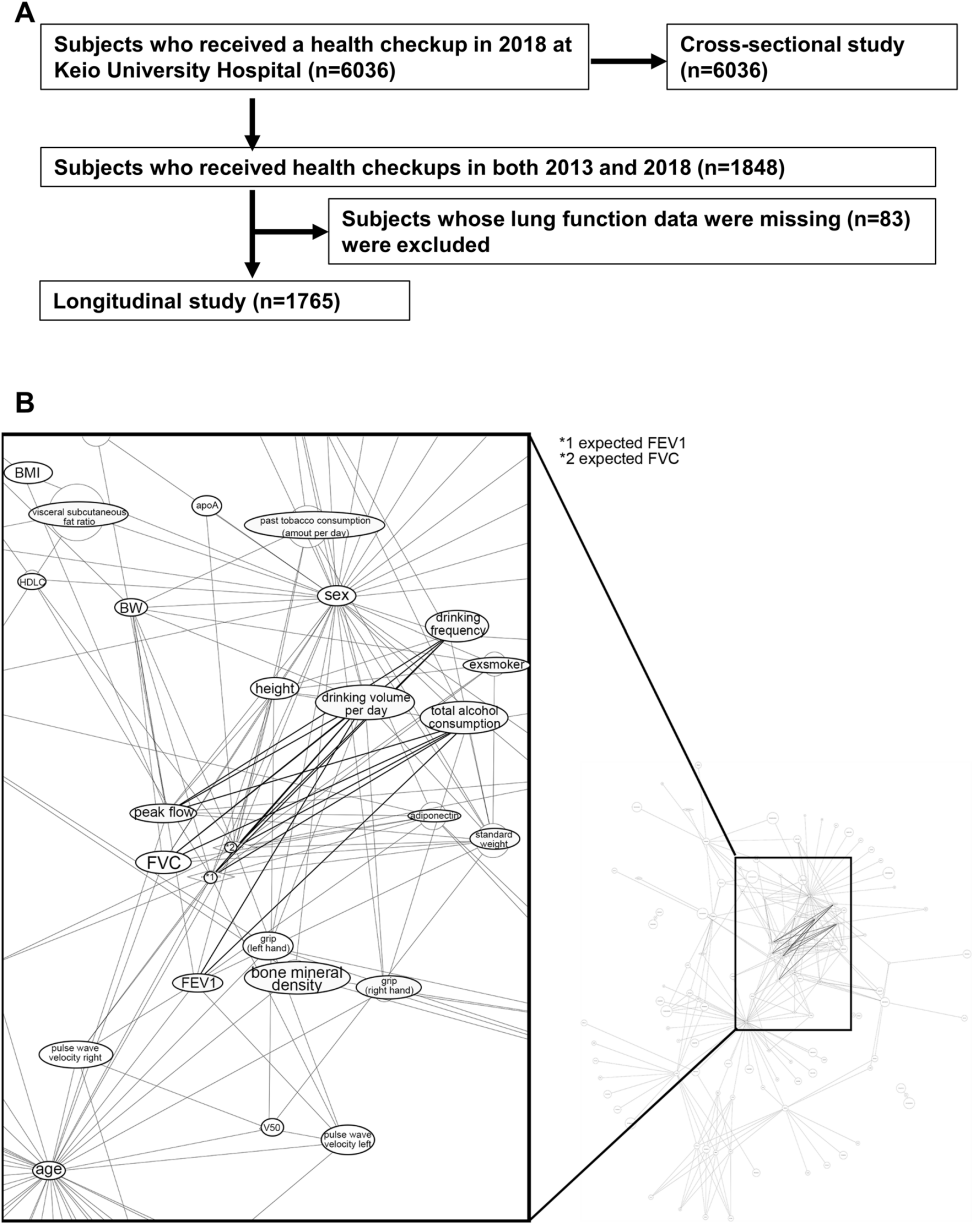
Comprehensive cross-sectional analysis and the subsequent longitudinal study using health checkup data. **(A)** Flow chart of the study population. 6036 subjects received health checkups in 2018, and their data were used for a cross-sectional study. Of these, 1848 subjects received health checkups in both 2013 and 2018, and 83 subjects, whose lung function data were missing, were excluded. Health checkup records of the remaining 1765 subjects were used for a longitudinal study. **(B)** Correlation network analysis by comprehensive PCIT shows the top 250 independent correlations between lifestyle questionnaire and health checkup data of subjects who received a health checkup in 2018. Well-established correlations were excluded. Correlation networks between drinking habits and lung function are highlighted. Graphviz version 2.26.0. software (https://graphviz.org/faq/) was used for drawing the diagram.

We reviewed health checkup records of 6036 adult Japanese who received health checkups in 2018 at Keio University Hospital and analyzed them in a cross-sectional study (Fig. 1A). Among 1848 subjects who received health checkups in both 2013 and 2018, 83 subjects whose lung function records were missing, were excluded, and health checkup records of the remaining 1765 subjects were used for a longitudinal study (Fig. 1A, Table 1). The average subject age was 60 years and 61.2% of the population was male (Table 1). Obese subjects with body mass index (BMI) more than 25 comprised only 25.6% of the total population (Table 1), which was slightly lower than the average Japanese population (32.2% in men, 21.9% in women; 34.6% of men in their 60 s, 27.5% of women) (Ministry of Health, Japanese National Health and Nutrition Survey, 2019). 49.5% of the subjects never smoked (Table 1). Those who never drank or heavy drinkers who drank more than 350 g per week were 26.4% and 8.7% (Table 1), respectively.

**Table 1:** Characteristics of subjects of the cross-sectional and longitudinal studies.

	Cross-sectional study (n = 6036)	Longitudinal study (n = 1765)	
	Year 2018	Year 2013	Year 2018
**Age**			
Mean (SD) (years)	60.0 (13.0)	61.3 (11.6)	
**Height**			
Mean (SD) (cm)	165.2 (8.8)	164.8 (8.6)	
**Sex**			
Male	61.2% (3696/6036)	65.0% (1148/1765)	
Female	38.8% (2340/6036)	35.0% (617/1765)	
**BMI**			
< 25	74.4% (4489/6036)	77.0% (1359/1765)	76.1% (1344/1765)
25–30	22.0% (1329/6036)	19.9% (351/1765)	20.4% (360/1765)
≥ 30	3.6% (218/6036)	3.1% (55/1765)	3.5% (61/1765)
**Smoking**			
Never	49.5% (2989/6036)	49.4% (871/1765)	47.5% (838/1765)
Previous	39.1% (2358/6036)	40.2% (709/1765)	43.6% (770/1765)
Current	10.6% (640/6036)	10.5% (185/1765)	8.2% (144/1765)
Data missing	0.8% (49/6036)	0% (0/1765)	0.7% (13/1765)
**Drinking**			
Never	26.4% (1596/6036)	26.5% (467/1765)	28.8% (508/1765)
Light	28.5% (1718/6036)	25.5% (450/1765)	26.9% (475/1765)
Moderate	36.0% (2175/6036)	37.2% (656/1765)	35.1% (620/1765)
Heavy	8.7% (524/6036)	8.8% (156/1765)	8.8% (155/1765)
Data missing	0.38% (23/6036)	2.0% (36/1765)	0.40% (7/1765)
**Spirometry (mean (SD))**			
FVC (L)	3.60 (0.90)	3.58 (0.86)	3.48 (0.88)
FEV1 (L)	2.71 (0.72)	2.71 (0.69)	2.55 (0.58)
FEV/FVC ratio (%)	75.4 (8.06)	75.7 (7.81)	73.5 (8.33)
Spirometory data missing	128	0	0

Characteristics (age, height, sex, BMI, smoking status, total alcohol consumption and spirometry) of subjects of the cross-sectional study who received health checkup in 2018 at Keio University Hospital and subjects of the longitudinal study who received health checkups at Keio University Hospital in 2013 and 2018.

### A cross-sectional study shows a positive correlation between moderate alcohol consumption and lung function

**Figure 2:**
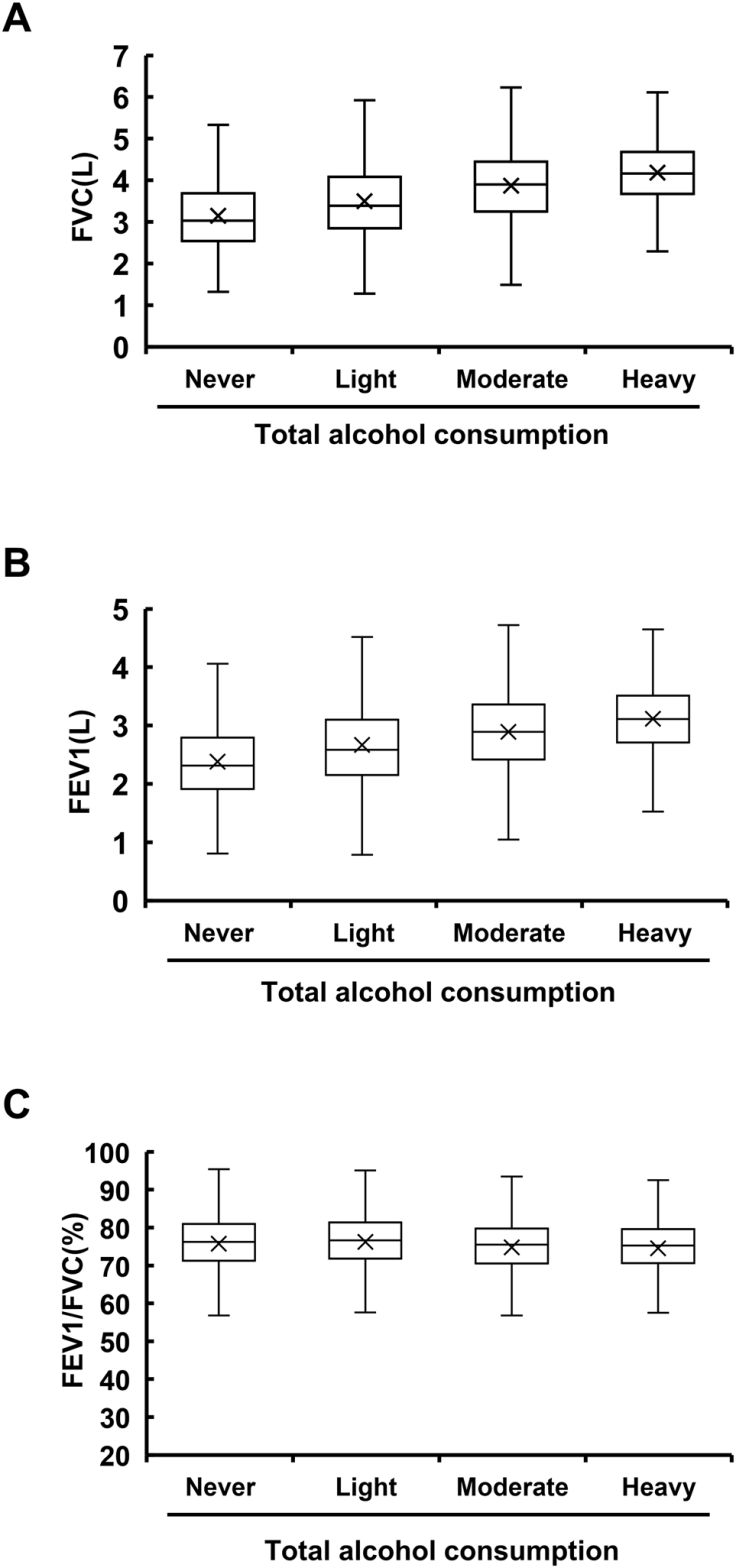
Lung function is positively correlated with alcohol consumption. **(A)** Box and whisker plot of FVC for each category of alcohol consumption. All subjects received a health checkup in 2018 at Keio University Hospital and categorized into 4 subgroups by total alcohol consumption as stated in Supplementary Table S6B. **(B)** Box and whisker plot of FEV1 for each category of alcohol consumption. **(C)** Box and whisker plot of the FEV1/FVC ratio for each category of alcohol consumption.

We applied unbiased, comprehensive correlation analysis to assess clinical data from 6036 patients in light of their responses to the lifestyle questionnaire. We first used correlation analysis and ANOVA to identify correlations with large coefficients and effect sizes. These included well-established correlations, such as a positive correlation of alcohol consumption with gamma GTP or uric acid, and a negative correlation of smoking with lung function, which we excluded from the list. We thoroughly investigated each correlation to discover novel impacts of lifestyle habits on clinical parameters. Especially, we focused on the effects of alcohol intake, many of which remain controversial. We unexpectedly found that moderate alcohol consumption was positively correlated with FVC and forced expiratory volume in one second (FEV1). Both correlation and ANOVA analysis showed a strong positive correlation of total alcohol consumption with FVC and FEV1 (Table 2). As a next step, we analyzed the data comprehensively using partial correlation and information theory (PCIT). This was originally designed to derive gene co-expression networks by identifying significant associations between expression profiles; however, we employed it to select only independent correlations that are not influenced by other confounding factors^3^. We applied PCIT analysis comprehensively to all variants obtained from the health checkup data and questionnaire, and independent correlations that PCIT defined as significant were listed according to the absolute value of the correlation coefficient. Then we excluded well-established correlations, and the top 250 correlation networks were selected (Fig. 1B). With PCIT, strong, positive, significant correlations were identified between total alcohol consumption and lung function (correlation value; FVC, 0.35; FEV1, 0.30) (Table 2). Indeed, from 2018 cross-sectional data, mean FVC and FEV1 were higher in groups with larger alcohol consumption in a dose-dependent manner (Fig. 2A,B), while no apparent difference in the FEV1/FVC ratio, a commonly used marker for obstructive pulmonary diseases^4^, was observed by the difference in total alcohol intake (Fig. 2C).

**Table 2:** An independent positive correlation between drinking habits and lung function calculated by three statistical algorithms.

	FVC			FEV1			FEV/FVC ratio		
	Total	Male	Female	Total	Male	Female	Total	Male	Female
**ANOVA**									
Drinking volume/day	0.70	0.61	0.62	0.69	0.60	0.61	− 0.50	0.53	0.53
Drinking frequency	0.66	0.56	0.58	0.64	0.55	0.57	− 0.50	− 0.50	− 0.50
Total alcohol consumption	0.67	0.58	0.59	0.65	0.57	0.58	− 0.50	0.50	0.51
**CORR**									
Drinking volume/day	0.40	0.21	0.23	0.37	0.20	0.23	− 0.04	0.06	0.07
Drinking frequency	0.32	0.13	0.17	0.27	0.10	0.14	− 0.08	− 0.01	− 0.00
Total alcohol consumption	0.35	0.16	0.17	0.30	0.14	0.16	− 0.07	0.004	0.03
**PCIT**									
Drinking volume/day	Sig	Sig	Sig	Sig	Sig	Sig	N.S.	N.S.	N.S.
Drinking frequency	Sig	N.S.	N.S.	Sig	N.S.	N.S.	N.S.	N.S.	N.S.
Total alcohol consumption	Sig	Sig	N.S.	Sig	N.S.	N.S.	N.S.	N.S.	N.S.

Correlation values between drinking habits and lung function calculated with three statistical algorithms (ANOVA, CORR, and PCIT) using data from subjects who received health checkups at Keio University Hospital in 2018 (n = 6036).

*Sig* significant, *N.S.* not significant.

### Longitudinal study shows that an increase in total alcohol consumption protects against time-related deterioration of FVC

**Figure 3:**
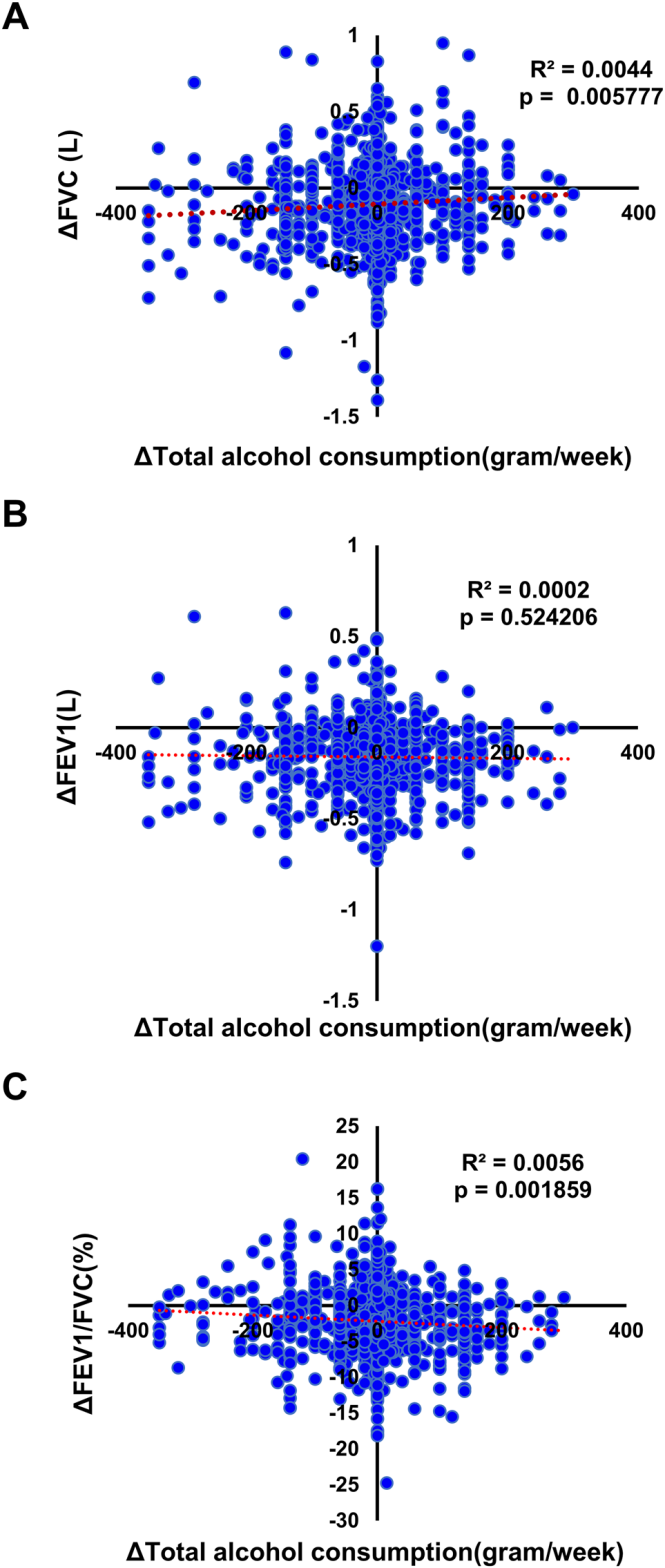
Increases in moderate alcohol consumption protect subjects against time-related physiological deterioration of FVC and FEV1, but not the FVC/FEV1 ratio. **(A)** Time course of the effect of changes in alcohol intake on time-related deterioration of FVC. The X axis represents the change in the total alcohol consumption and the Y axis represents the change in FVC from the same persons in 2013 and 2018 in all subplots. The red dotted line represents an approximate straight line. **(B)** Time course analysis of the effect of changes in alcohol intake on time-related deterioration of FEV1. **(C)** Time course analysis of the effect of changes in alcohol intake on time-related deterioration of the FEV1/FVC ratio.

The effect of alcohol consumption and lung function has remained controversial. Therefore, we analyzed the impact of a temporal change in alcohol consumption on time-related physiological deterioration of lung function. We compared health checkup data of the same persons who received health checkups in 2013 and 2018 at Keio University Hospital (n = 1765) (Fig. 1A). Characteristics of subjects in this longitudinal study were comparable to those in the cross-sectional study of 2018 (Table 1). Lung function of the same persons, including FVC and FEV1, declined over time (Fig. 3A,B). From 2013 to 2018, 53.3% of the subjects did not change total alcohol consumption, but 34.8% of the subjects either increased or decreased alcohol intake by less than 100 g/week and 11.9% changed alcohol intake by more than 100 g/week (Supplementary Table S1). Surprisingly, the increase in total alcohol consumption over 5 years significantly attenuated time-related FVC deterioration in a dose-dependent manner (*P* = 0.0058) (Fig. 3A), consistent with the strong positive correlation between FVC and alcohol intake observed in the single-year, cross-sectional study described above (Table 2, Fig. 2A).

We also analyzed the effect of alcohol consumption change on FEV1 over time, but in contrast to the strong positive impact of alcohol consumption on FVC over time, no significant relationship was observed between FEV1 decline over time and a change in total alcohol consumption (*P* = 0.52) (Fig. 3B).

Although the FEV1/FVC ratio did not show a significant direct correlation with alcohol consumption in the cross-sectional study by PCIT (Table 2), we analyzed the effect of changed alcohol intake over time on the FEV1/FVC ratio using data from 2013 and 2018. The FEV1/FVC ratio declined over time (Fig. 3C), and as expected from the lack of significant impact of alcohol intake on FEV1 (the numerator), and the strong positive effect of alcohol consumption on FVC (the denominator), the decline of FEV1/FVC from 2013 to 2018 showed a significant negative linear correlation with the change in total alcohol intake from 2013 to 2018 (*P* = 0.0019) (Fig. 3C).

### Higher baseline alcohol consumption maintains larger FVC and FEV1 in subjects who did not change drinking habits during the 5-year study

**Figure 4:**
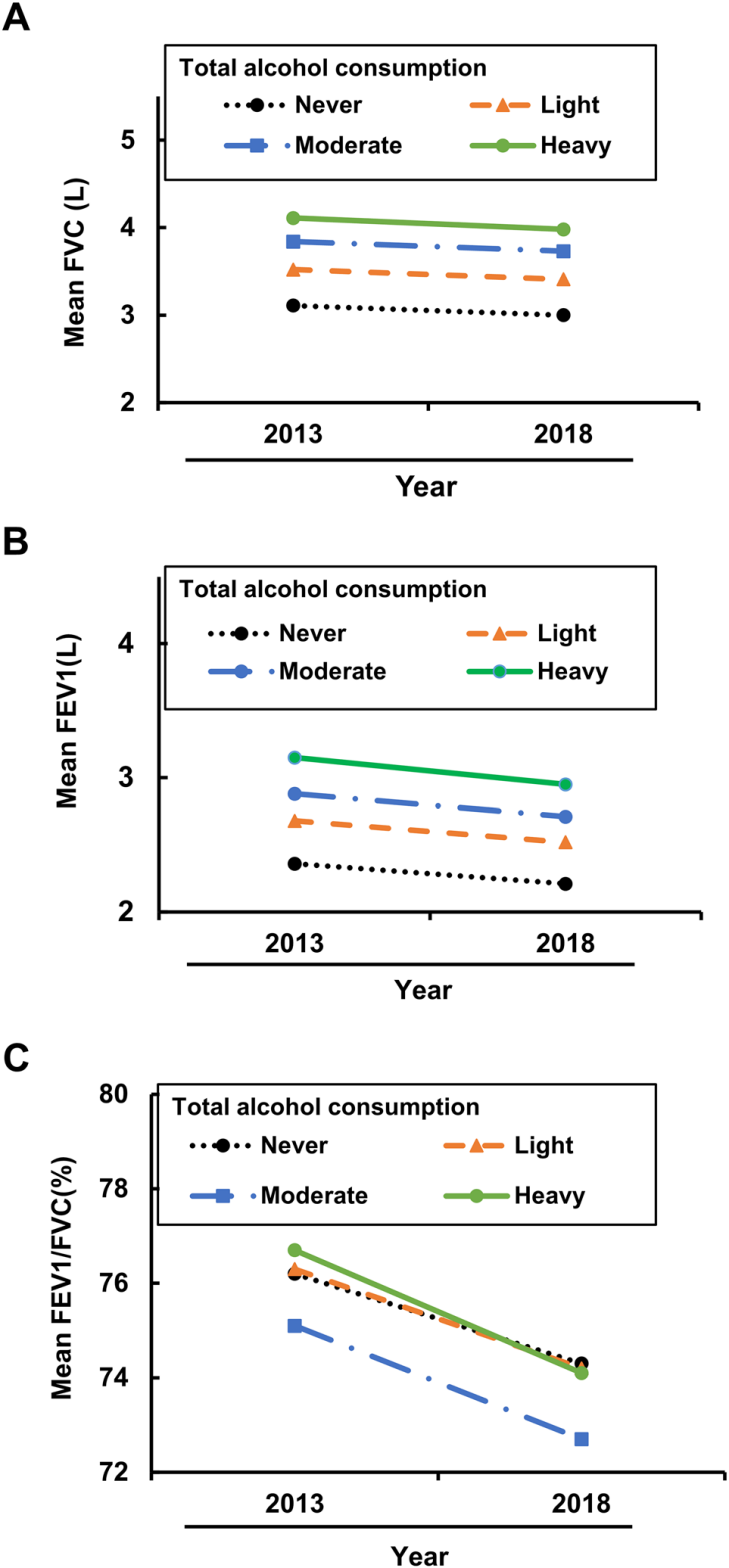
Greater baseline alcohol consumption maintains larger FVC and FEV1 in subjects who did not change their drinking habits during the 5-year study. Subjects were divided into four groups according to their alcohol intake as stated in Supplementary Table S6B, and only those subjects whose category did not change from 2013 to 2018 were analyzed. **(A)** The mean FVC value in each category of total alcohol intake in 2013 and 2018. Black circles represent never drinkers (n = 411), orange triangles represent light drinkers (n = 327), blue circles represent moderate drinkers (n = 503), green circles represent heavy drinkers (n = 99). These symbols apply to all subplots. 383 subjects were omitted. **(B)** The mean FEV1 value in each category of alcohol intake in 2013 and 2018. **(C)** The mean FEV1/FVC ratio value in each category of alcohol intake in 2013 and 2018.

Since major changes in drinking habits could reflect subjects who quit drinking because of alcohol-related or other medical problems, we re-categorized the longitudinal study population according to the degree of total alcohol consumption into 4 subgroups. We omitted 383 subjects whose alcohol intake category changed between 2013 and 2018. Among the remaining subjects, we analyzed the change in lung function for each category. We found a similar trend to that in the cross-sectional study. Subjects with a higher baseline alcohol consumption maintained larger FVC and FEV1 after 5 years in a dose-dependent manner (Fig. 4A,B). As for FEV1/FVC ratio, moderate drinkers showed a lower value over 5 years, but the difference between non-drinkers and light drinkers and relatively heavy drinkers was not clear (Fig. 4C).

### Men show a stronger effect of alcohol consumption on lung function than women

Alcohol consumption habits differ greatly between men and women^5^. Thus, we first examined the effect of alcohol on lung function cross-sectionally, dividing the data by gender. Total alcohol consumption was significantly lower among women (58.6% of men and 22.8% of women were classified as moderate to heavy drinkers), whereas 41.8% of women and 16.8% of men did not drink at all (Supplementary Table S2). Although significant independent correlations between alcohol consumption and FVC, FEV1 were observed in the PCIT analysis of all subjects of the cross-sectional study (alcohol and FVC; correlation value = 0.35, alcohol and FEV1; correlation value = 0.30), when gender-segregated, the independent significant associations of alcohol intake and FVC were limited to the male population (correlation value = male 0.16, female N.D.), and no significant association was observed between alcohol intake and FEV1 by PCIT (Table 2).

Next, we examined the effect of alcohol on lung function temporally, dividing the data by gender. As with the overall data, an increase in alcohol consumption significantly attenuated the decline in FVC (*P* = 0.019) (Supplementary Fig. S1A) and exacerbated the decline in the FEV/FVC1 ratio (*P* = 0.0086) (Supplementary Fig. S1B) from 2013 to 2018 among men.

Among women, in addition to the reduced tendency to drink, there was smaller change in drinking habits than among men from 2013 to 2018 (Supplementary Table S1). Unlike men, no significant correlation between the change in alcohol intake and the change in either FVC or FEV1/FVC ratio was observed in women. (Supplementary Fig. S1C,D).

### The temporal effect of alcohol consumption on lung function is independent of smoking

Alcohol intake is strongly connected with smoking, which is detrimental to lung function^6^. The percentage of smokers among non-drinkers was only 6.2%, while the percentage of smokers among drinkers was 12.3% (Supplementary Table S3A). From the cross-sectional study, the PCIT algorithm defined the association of alcohol intake and lung function as significantly direct, even excluding the potential confounder smoking effect. We also analyzed the effect of a change in tobacco consumption per day on the decline in FVC and FEV1/FVC from 2013 to 2018. We found that 86.2% of subjects did not change smoking habits, but 9.5% of subjects either increased or decreased the consumption of tobacco by 5–10 cigarettes /day and 4.32% changed by more than 10 cigarettes/day (Supplementary Table S3B). Surprisingly, there was no apparent trend between the change in tobacco consumption per day and the decline in FVC or FEV1/FVC ratio during this 5-year study (Supplementary Fig. S2A,B). Taken together, the positive effect of alcohol intake on FVC and the negative effect on FEV1/FVC ratio are independent of smoking habits, both cross-sectionally and longitudinally.

### A change in alcohol consumption does not affect the time-related decline in total lung volume

**Figure 5:**
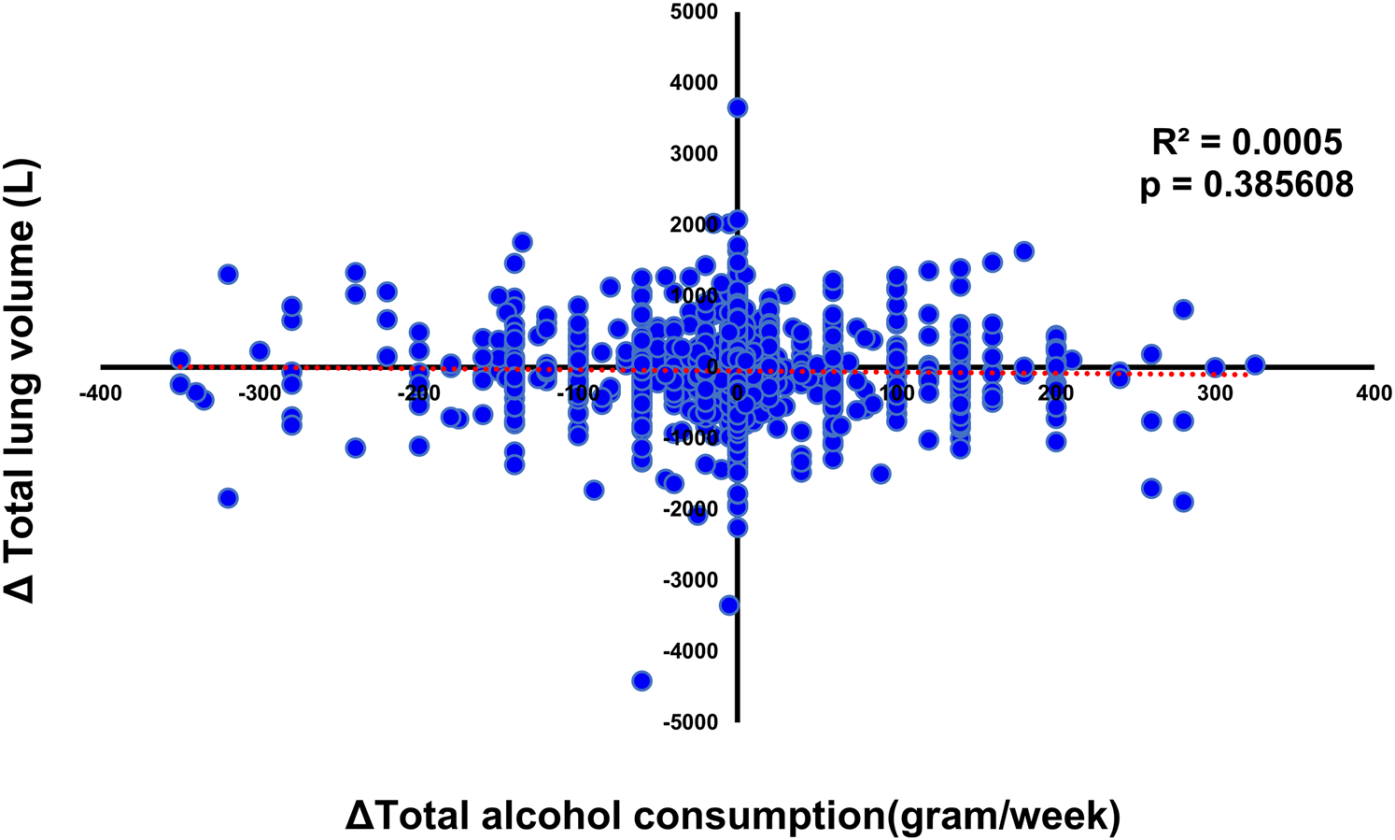
Changes in alcohol consumption did not affect time-related total lung volume decline. Time course analysis of the effect of changes in alcohol intake on time-related deterioration of total lung volume calculated from CT scans. The X axis represents the change in the alcohol intake and the Y axis represents changes in total lung volume from the same persons in 2013 and 2018. The line represents an approximate straight line.

Since it was clear that alcohol consumption affects lung function, we next compared physical lung volume of the same persons in 2013 and 2018, as measured by CT scans included in the health checkup data. Lung volume deteriorated over time, but the change in total alcohol consumption did not significantly affect lung volume (*P* = 0.39) (Fig. 5).

### The positive effect of alcohol on skeletal muscle mass and grip, which may reflect respiratory muscle mass, may explain the protective effect of moderate alcohol consumption on FVC

**Figure 6:**
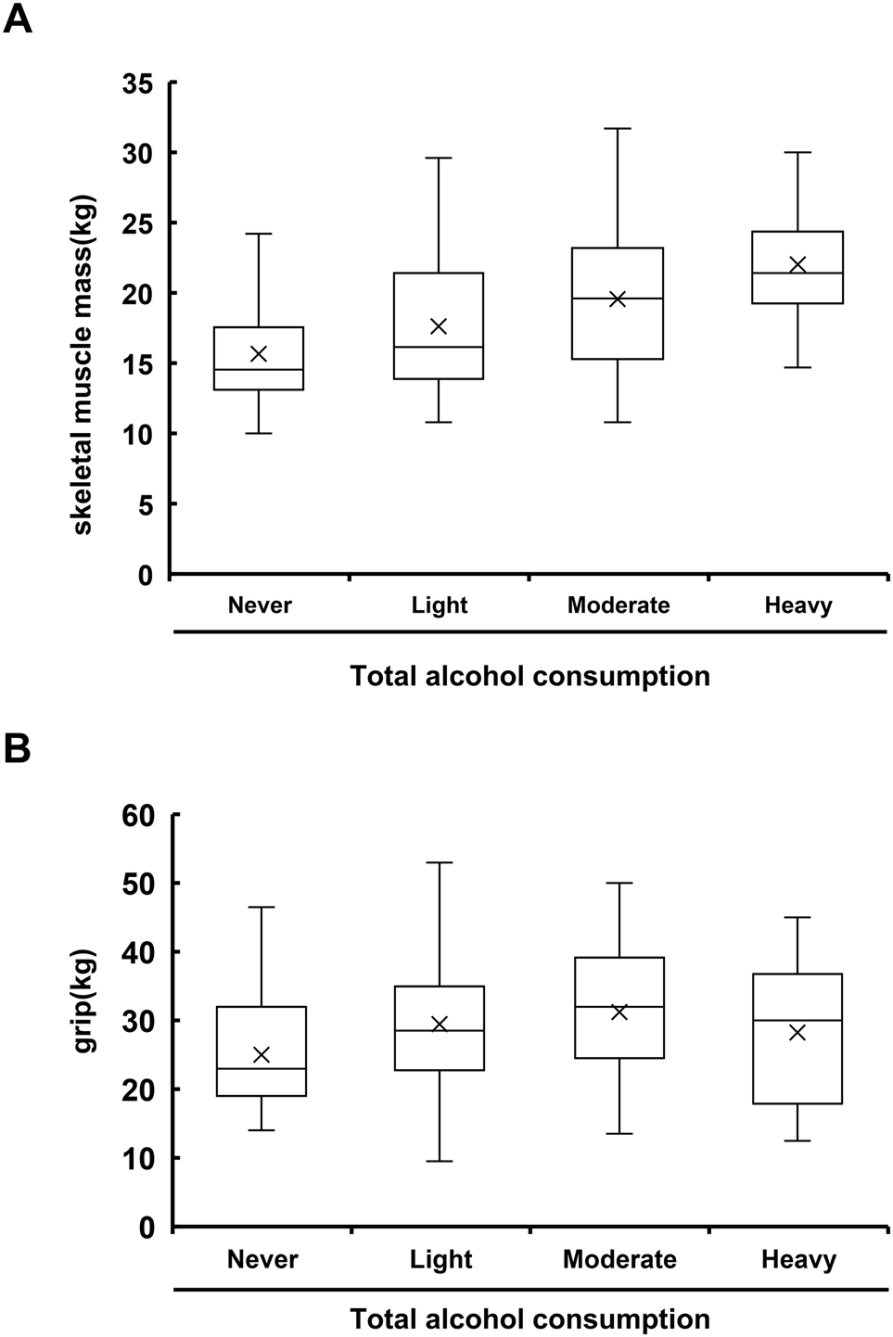
Skeletal muscle mass/strength is positively correlated with alcohol consumption. **(A)** Box and whisker plot of skeletal muscle mass for each category of alcohol intake from subjects who received health checkups in 2018 at Keio University Hospital. **(B)** Box and whisker plot of grip for each category of alcohol consumption.

Since a major effect of alcohol on lung function is evident in FVC protection without affecting lung volume, we hypothesized that respiratory muscle volume/strength may be affected by alcohol intake. An association between skeletal muscle mass/strength and respiratory muscle strength and lung function, including FVC in healthy populations^7–12^, has been reported. Also, previous studies showed that age-related decreases in skeletal muscle mass/strength, such as sarcopenia, are associated with decreased respiratory muscle strength and lung function in chronic respiratory diseases in the elderly^13–16^. These data suggest that skeletal muscle mass and grip may reflect respiratory muscle volume and strength. To date, a relationship between alcohol consumption and skeletal muscle mass/strength has not been reported. We examined the correlation between alcohol intake and skeletal muscle mass/grip using cross-sectional data. PCIT analysis defined the positive correlations between total alcohol consumption and skeletal muscle mass/grip as significantly direct and these associations are listed among the 250 correlation networks (correlation value; alcohol and skeletal muscle mass, 0.39; alcohol and grip, 0.30) (Supplementary Table S4). Strong positive correlations between alcohol intake and skeletal muscle mass/grip were also confirmed by correlation analysis and ANOVA (Supplementary Table S4). From the 2018 cross-sectional data, skeletal muscle mass is higher in individuals who consumed more alcohol (Fig. 6A), and grip, markers of skeletal muscle strength, was also higher in light to moderate drinkers than non-drinkers (Fig. 6B). We further analyzed whether drinking habits affect exercise, including walking speed, but PCIT showed no direct association between total alcohol intake and exercise or walking speed.

### An anti-inflammatory effect of alcohol was confirmed longitudinally, but the magnitude was small

As another possible mechanism of the protective effect of alcohol on lung function, we considered its possible anti-inflammatory activity^17^. We examined the correlation of C-reactive protein (CRP) as a systemic inflammatory marker and drinking habits, using our cross-sectional study data. Although PCIT analysis of the cross-sectional study showed no significant independent correlation between total alcohol intake and CRP, we confirmed a small, but significant negative longitudinal correlation between alcohol consumption and CRP (*P* = 0.038) (Supplementary Fig. S3A). White blood cell count was not affected by a change in drinking (Supplementary Fig. S3B).

### Drinking amount per day is more critical than drinking frequency in relation to lung function

Recent studies have suggested the importance of drinking pattern^18^, especially the strong negative impact of binge drinking^19,20^. Our method cannot distinguish a person who drinks 40–60 g/day, 1–2 days per week from a person who drinks < 20 g/day for 5–6 days. We further analyzed the effect of alcohol on lung function, separating the drinking amount at one time and drinking frequency for both cross-sectional and longitudinal studies. PCIT analysis of the cross-sectional data from 2018 showed significant independent positive correlations of FVC, FEV1 and both drinking amount at one time and drinking frequency, but the strength of correlation was stronger with drinking amount at one time than with drinking frequency (correlation value; FVC and drinking volume per day, 0.40; FEV1 and drinking volume per day, 0.37; FVC and drinking frequency, 0.32; FEV1 and drinking frequency, 0.27) (Table 2). The longitudinal study showed that an increase in drinking amount at one time significantly protected against time-related deterioration of FVC (*P* = 0.017), while the change in drinking frequency over time did not significantly affect temporal FVC decline (*P* = 0.23) (Supplementary Fig. S4A,B). The significant negative linear correlation with the temporal decline in FEV1/FVC ratio and the change in total alcohol intake was also preserved when analyzed with the change in the drinking amount at one time over time (*P* = 0.014), but the changes in drinking frequency did not affect the time-related deterioration of FEV1/FVC ratio (*P* = 0.15) (Supplementary Fig. S4C,D). Neither the change in drinking amount at one time nor drinking frequency affected time-related deterioration of FEV1 significantly (FEV1 and drinking volume per day, *P* = 0.89; FEV1 and drinking frequency, *P* = 0.91) (Supplementary Fig. S4E,F). Taken together, these results suggest that drinking volume at one time is more critical than drinking frequency in relation to lung function.

## Discussion

Using comprehensive partial correlation network analysis of 6036 subjects in a cross-sectional study, we found positive correlations between lung function and low-dose alcohol consumption, which were also correlated with muscle mass and strength. We further confirmed that a significant increase in mild to moderate alcohol intake attenuated the age-related decline of FVC, in a longitudinal study comparing data from the same subjects. On the other hand, the decline in FEV1/FVC ratio was exacerbated by increased total alcohol intake, and lung volume, measured by CT scan images, was not significantly affected by a change in total alcohol consumption. Among subjects who did not change drinking habits, higher baseline alcohol consumption maintained larger FVC and FEV1 with a smaller FEV1/FVC ratio over 5 years.

The most important feature of our study is that we started from a comprehensive cross-sectional analysis of a large dataset using a statistical approach that avoided bias to discover a novel, independent impact of moderate alcohol consumption on health. In view of results showing a positive effect of alcohol intake on lung function, which has been a topic of conflicting reports^21–24^, we developed an analysis based upon health checkup data collected longitudinally from the same persons. Hence, our study started as a data-driven, hypothesis-generating study, and developed into a hypothesis-testing, traditional epidemiological study.

We applied partial correlation and information theory comprehensively to all variables from the health checkup data to find independent correlations between life habits and physical data. Originally, the PCIT algorithm was developed to identify small but significant correlations that would be overlooked by the traditional constant cutoff approach in building gene co-expression networks^3^. Previous studies using the PCIT algorithm have mostly been limited to genetic research^3,25^. However, in theory, PCIT can be applied to routine clinical data to test correlations and the significance threshold of all clinical parameters, including life-style habits. Analysis of clinical data is always complicated by confounding factors, and PCIT has the strength of finding unexpected weak, but significant direct correlations between clinical elements, sequentially taking into account a large number of potential confounders compared to traditional approaches.

Most previous studies, including both clinical cross-sectional studies and basic research on the effect of alcohol on lung function, have highlighted its negative effects^26–29^. However, these previous studies focused on heavy drinkers, including alcoholic patients and alcohol volumes sufficient to induce intoxication. Interpretation of lung function parameters from heavy drinkers has also been complicated by a close association with heavy smoking^6^. Some past studies of alcohol effects have included but failed to account for subjects consuming varied amounts of alcohol^22,30^. Recent reports that target light to moderate drinkers have shown enhanced lung function in relation to alcohol intake^17,31–34^. Our study population consisted of subjects whose alcohol consumption is mostly light to moderate with no attrition. Most of the past studies targeting mild drinkers assessed lifestyle habits only at baseline and did not examine changes in these habits during the study period^17,31–34^. In this study, we succeeded in analyzing temporal effects of changes in drinking habits on lung function using health checkup data that recorded both physical data and lifestyle data from the same persons longitudinally.

Annual health checkup data that we employed included a detailed lifestyle questionnaire, which enabled us to analyze the differential effect of alcohol volume consumed per day and drinking frequency. Many past studies have reported that mild to moderate drinking positively affects heart disease^35–37^, and recently these have focused on the importance of drinking pattern^18^. Recently, the strong negative impact of binge drinking has attracted attention to toxic effects of alcohol^20,38^. Our data suggest for the first time that drinking amount at one time is more critical than drinking frequency, in relation to lung function.

Alcohol consumption habits differ greatly between men and women^5^. Thus, we examined the effect of alcohol on lung function both cross-sectionally and longitudinally, dividing the data by gender and found that the effect is limited only to men. Our study population comprised approximately 1.8-fold more men than women. Also, the proportion of those who never drink was 41.8% for women vs 10.8% for men. These factors might have affected the gender difference.

Our study showed a positive association between alcohol and FVC, but the effect of alcohol on the FEV1/FVC ratio was negative. From a clinical standpoint, it is complicated to interpret this data. The increase in FVC due to increased alcohol intake suggests a protective effect of alcohol on spirometric restrictive disorders. The FEV1/FVC ratio is generally used as a conventional marker to discern spirometric obstructive disorders^4^, but the decreased FEV1/FVC ratio cannot be interpreted simply as an increased risk of obstructive pulmonary disorders. Our present data include only FVC, but do not include simple VC. For that purpose, we analyzed lung volume longitudinally using CT scan data instead. Lung volume closely reflects total lung capacity, although the two are not exactly equivalent^39–41^, and our data showed that long-term, lung volume was not affected by changes in alcohol intake. In restrictive pulmonary disorders such as COPD, total lung capacity increases by lung hyperinflation while FVC decreases, resulting in an increase of residual volume^42,43^. We speculate from non-affected lung volume and increased FVC evident in our data that alcohol might decrease residual volume, suggesting a possible protective effect of alcohol on COPD. Indeed, a retrospective autopsy study among male veterans showed an inverse relationship of alcohol consumption to emphysema^44^. However, alcohol intake is a potent risk factor for other social and medical problems, including various cancers and injury^45^, and in actual clinical settings, the net benefit to the patient should be carefully considered in terms of alcohol consumption.

The possible protective effect of alcohol on lung function may be attributable to multiple mechanisms, but from our cross-sectional study we found that alcohol has a positive effect on muscle mass and strength, and this increase in skeletal muscle mass by alcohol may explain the increase in FVC by drinking, observed in this study. Sarcopenia is associated with decreased respiratory muscle strength and lung function, including FVC, in the elderly and in patients with chronic respiratory diseases^13–16^. Moreover, skeletal muscle mass/strength and respiratory muscle strength and lung function, including FVC, are likewise associated in healthy people^7–12^. Taken together, decreases in peripheral muscle mass and strength may reflect changes in the mass and strength of respiratory muscle, which directly affects FVC. Our data suggest the possibility that low-dose alcohol may increase respiratory muscle mass and strength without affecting total lung volume, leading to an increase in FVC.

There are several clinical studies showing small alcohol intake is accompanied by decreased inflammatory markers such as CRP and fibrinogen, indicating a possibility that small alcohol intake might decrease systemic inflammation^17^. Although the PCIT analysis of our cross-sectional study showed no independent association between CRP and alcohol intake, in the longitudinal analysis, we confirmed a small, but significant negative correlation between alcohol intake and CRP. White blood cell count was not affected by drinking habit changes, suggesting that this possible anti-inflammatory effect of alcohol is chronic and of small magnitude.

Our study has several limitations related to data and analytical methods. Although the PCIT analytical method investigated all measured variables, there could be unmeasured latent variables in the health checkup data. PCIT analysis is based on partial correlations of three factors. Theoretically, this method excludes the effects of single confounding factors from a correlation of two factors, but cannot evaluate the joint effect of multiple confounding factors. Therefore, we confirmed our findings from the PCIT analysis of the cross-sectional data by 5-year longitudinal study in the same persons. In addition, there may be a social-desirability bias that underreports alcohol consumption, since the data on alcohol consumption were based on a self-completed questionnaire by the subjects. Bias toward reverse causality stemming from patient illness, should be taken into account. This issue has been discussed for studies that show lower risk of coronary artery disease among light to moderate drinkers than among non-drinkers^46^. Our longitudinal study may have included subjects who quit drinking due to illness. However, when analyzing subjects who increased habitual drinking, the positive effect of alcohol intake was apparent. We also examined the temporal impact of alcohol intake, focusing on subjects whose alcohol intake did not change. We further found a positive effect of alcohol intake on FVC and FEV1, showing that this effect was substantial and unaffected by those who quit drinking due to illness. As for other limitations, most of the participants in our study are more health-conscious than average, and the tendency is likely more pronounced among participants in the longitudinal study who repeatedly attended health checkups. This health-consciousness might have affected the analysis. The small percentage of heavy drinkers, especially in the longitudinal study, may have lessened the influence of temporal alcohol-induced attrition, including lung function. All examinations in the health checkups were performed by experienced health professionals, but some data such as spirometric measurements may have been derived from imperfect measurements, because subjects could not follow instructions. Moreover, our data were derived from a single hospital, and to generalize our findings, data collection from multiple facilities is desirable.

In conclusion, we introduced a novel approach to the analysis of health checkup data cross-sectionally, using PCIT algorithm to identify previously missed patterns of association that may indicate as-yet uninvestigated causal pathways in an unbiased fashion. By further confirming the associations longitudinally, we found a possible positive effect of alcohol consumption on FVC without an effect on total lung volume, a trend that was independent of smoking.

## Methods

### Study assessments

The health checkup included a standard questionnaire, a physical examination, biochemical tests, abdominal ultrasonography, and a chest CT scan (Supplementary Table S5). The questionnaire assessed each subject’s medical history, as well as lifestyle habits, including alcohol consumption, smoking, and exercise, and was self-completed by the subjects. Questions and the range of patient responses are shown in Supplementary Table S5. Height and weight were measured without shoes or heavy clothing, and BMI was calculated. Spirometry data was obtained with a spirometer (CHESTAC-8900, Chest M.I., Tokyo, Japan), according to international recommendations^47,48^. Muscle volume was measured as appendicular skeletal muscle mass, which was defined as the lean body mass of both the upper and lower extremities^49^. The lean body mass was scanned using whole-body DEXA scans (LUNAR PRODIGY Advance; GE Healthcare Japan Corporation, Tokyo, Japan) with enCORE software, version 9.2 (GE Healthcare)^49^. Unenhanced chest CT examinations were performed on a scanner (AQUILION One, CANON Medical Systems, Otawara, Japan) in the supine position with arms down during deep inspiration breath-hold to take axial images of 2-mm slice thickness^48^. All medical measurements and tests were performed by experienced health professionals. All persons who received health checkups in 2018 at Keio University Hospital were recruited into this study by obtaining informed consent in the form of an opt-out on the website of Keio University Hospital. The study protocol, including the method of opt-out consent, was approved by the Institutional Review Board at Keio University School of Medicine (20190254), and the study was conducted in accordance with concepts outlined in the Declaration of Helsinki.

### Quantitative drinking categories

For evaluation of alcohol exposure, self-completed answers to two types of questions, “how much do you drink?” accompanied by a choice of volume categories (e.g. < 20 g/day, 21-40 g/day…), and “how often do you drink?” accompanied by a choice of frequency categories (e.g., a few times per month, 1–2 days per week, 3–4 days per week…) were used. We calculated total alcohol consumption per week by multiplying the mean level of each answer category from each subject, combining both volume per day and frequency for the analysis (Supplementary Table S6A). In some analysis, study population was categorized into 4 subgroups, (1) never drinker, (2) light drinker, (3) moderate drinker, (4) heavy drinker, according to the degree of total alcohol consumption (Supplementary Table S6B).

### Total lung volume measurement

Total lung volume was measured using a deep learning model, U-net (R231) model, based upon chest CT scan images taken in 2013 and 2018 from the same subjects. This model was trained on a large, diverse dataset that covers a wide range of image variability and performs segmentation on individual slices, extracts right and left lungs separately, and includes air pockets, tumors, and effusions, excluding tracheae^50^.

### Cross-sectional statistical analysis

For correlation analysis and ANOVA, correlation coefficients were calculated using Excel. For PCIT, we wrote an original program as follows using FORTRUN as a coding language based on the previously described formula^3^. Briefly, for every trio of items listed in health checkup data in *x,y* and *z*, the three first-order partial correlation coefficients were computed by: r_*xy,z*_ = (r_*xy*_-r_*xz*_ r_*yz*_)/(SQRT (1–r^2^_*xz*_) (1–r^2^_*yz*_)) and similarly for r_*xz,y*_ and r_*yz,x*_. Then, to derive the tolerance level (ɛ) to be used as the local threshold for capturing significant associations, the average ratio of partial to direct correlation was computed as follows: ɛ = 1/3 (r_*xy,z*_/r_*xy*_ + r_*xz,y*_/r_*xz*_ + r_*yz,x*_/r_*yz*_). A connection between items *x* and *y* was discarded if: ∣r_*xy*_∣ ≤ ∣ɛ r_*xz*_∣ and ∣r_*xy*_∣ ≤ ∣ɛ r_*yz*_∣.Otherwise, the association was defined as significant, and to ascertain the significance of association between items *x* and *y*, the above-mentioned procedures were repeated for each of the remaining *n*-2 items (denoted here by *z*). We further applied this program comprehensively to all variables obtained from the 2018 cross-sectional data. For drawing a correlation network diagram, we used Graphviz version 2.26.0 software (https://graphviz.org/faq/)^51^.

### Longitudinal statistical analysis

Correlation efficient (R) were calculated using Excel. Degrees of freedom were defined as “sample number”-2. T-value was defined as ABS (R, *SQRT (degree of freedom)/SQRT (1 − R^2^)). P-values were calculated as TDIST (t-value, degrees of freedom, 2). A value of *P* < 0.05 and the absolute value of R > 0.05 was considered statistically significant.

## Supplementary Information

Supplementary Figures.

Supplementary Tables.
