# Supplementary material for: Unbiased, comprehensive analysis of Japanese health checkup data reveals a protective effect of light to moderate alcohol consumption on lung function
Source: Sci Rep. 2021 Aug 5;11:15954. doi: 10.1038/s41598-021-95515-4 (PMC8342527; doi:10.1038/s41598-021-95515-4)
Supplement: Supplementary file 1 — Supplementary Figures. [file 41598_2021_95515_MOESM1_ESM.docx]

**
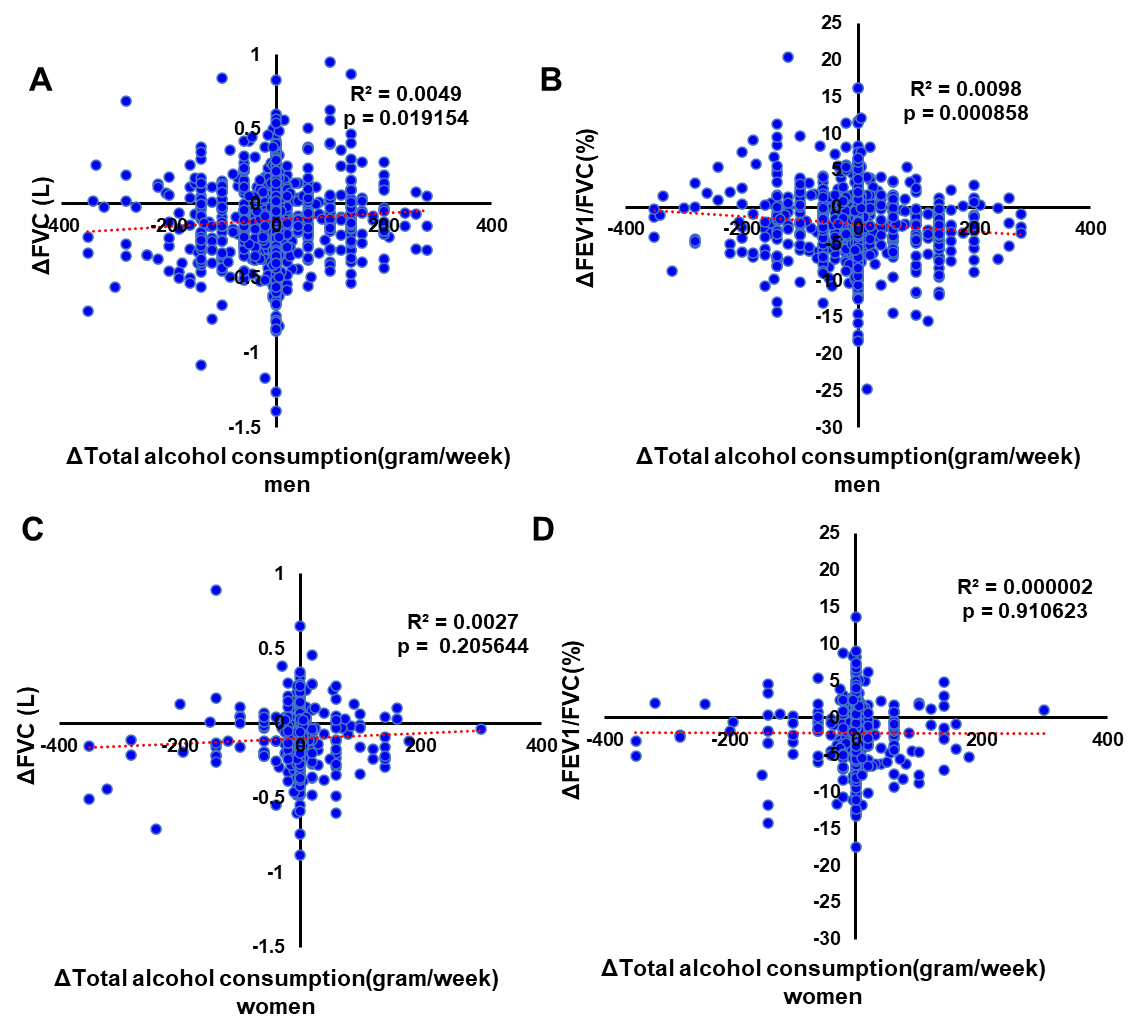
**

**Supplementary Fig. S1. Gender segregated longitudinal effect of alcohol consumption on lung function.** The red dotted line in each figure represents approximate straight line. **A**, Time course analysis of the effect of changes in total alcohol consumption on time-related deterioration of FVC in men. The X-axis represents changes in the alcohol consumption and the Y-axis represents changes in FVC from the same men in 2013 and 2018. **B**, Time course analysis of the effect of changes in alcohol intake on time-related deterioration of the FEV1/FVC ratio in men. The X-axis represents changes in the alcohol intake and the Y-axis represents changes in the FEV1/FVC ratio from the same men in 2013 and 2018. **C**, Time course analysis of the effect of changes in alcohol intake on time-related deterioration of FVC in women. The X-axis represents changes in the alcohol intake and the Y-axis represents changes in FVC from the same women in 2013 and 2018. **D**, Time course analysis of the effect of changes in alcohol intake on time-related deterioration of FEV1/FVC ratio in women. The X-axis represents changes in alcohol intake and the Y-axis represents changes in FEV1/FVC ratio from the same women in 2013 and 2018.


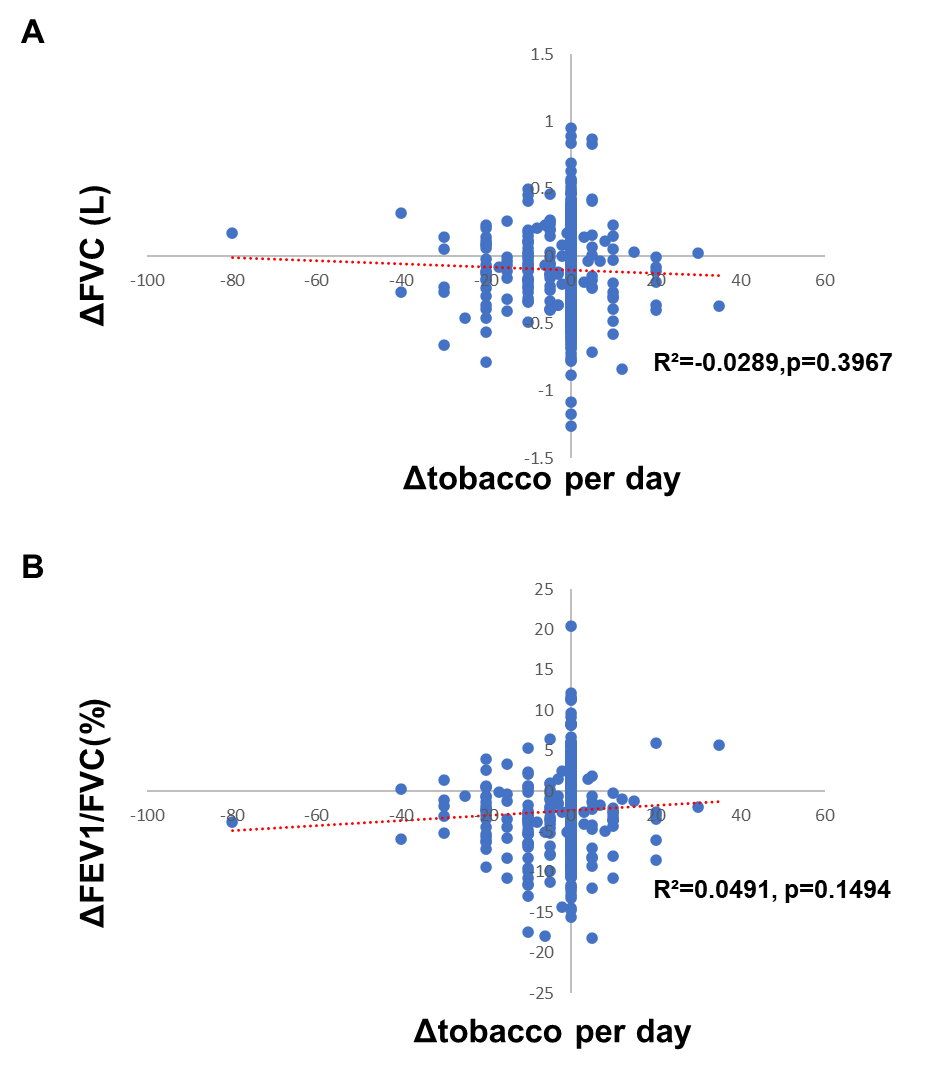


**Supplementary Fig. S2. The effect of changes in daily tobacco consumption on time related physiological deterioration of lung function. A**, Time course analysis of the effect of changes in tobacco consumption per day on time-related deterioration of FVC. The X axis represents changes in tobacco consumption per day and the Y-axis represents changes in FVC from the same persons in 2013 and 2018. The red dotted line represents approximate straight line. **B**, Time course analysis of the effect of changes in tobacco consumption per day on time-related deterioration of the FEV1/FVC ratio. The X-axis represents changes in tobacco consumption per day and the Y-axis represents changes in the FEV1/FVC ratio from the same persons in 2013 and 2018. The red dotted line represents approximate straight line.


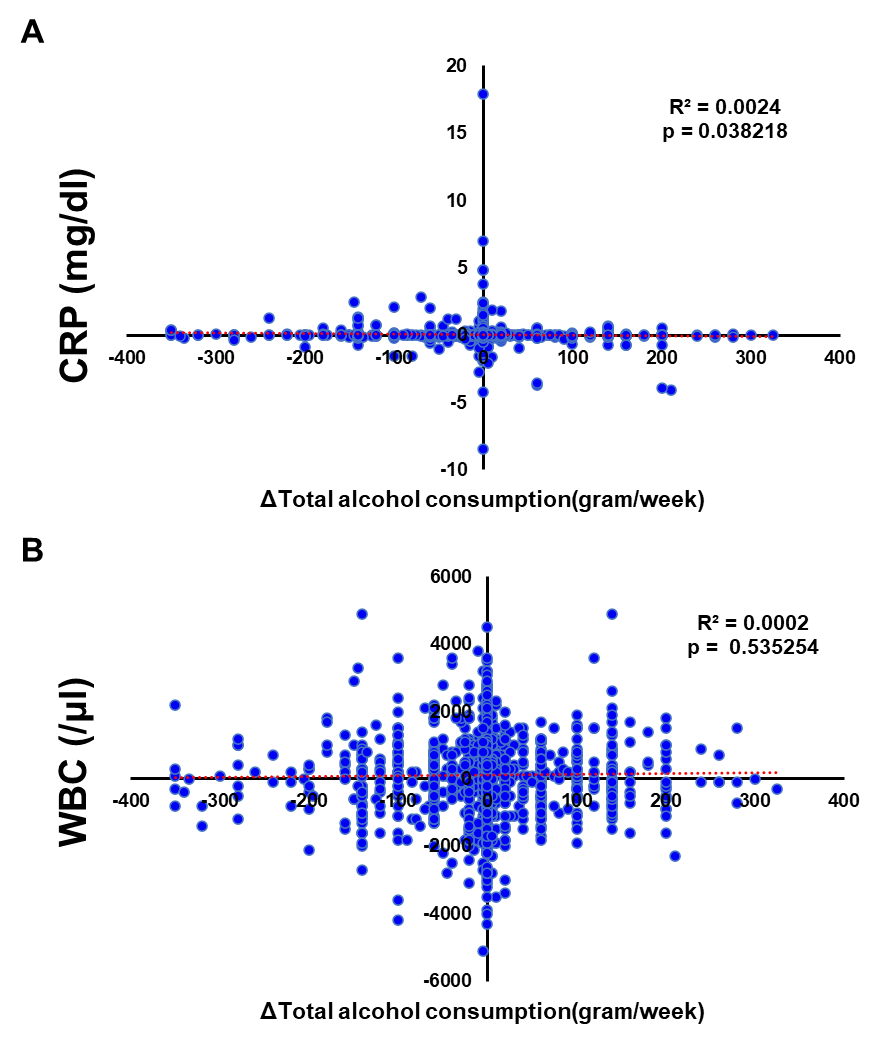


**Supplementary Fig. S3. The effect of changes in total alcohol consumption on inflammatory markers. A**, Time course analysis of the effect of changes in alcohol intake on CRP. The X-axis represents changes in alcohol intake and the Y-axis represents changes in CRP from the same persons in 2013 and 2018. The red dotted line represents approximate straight line. **B**, Time course analysis of the effect of changes in alcohol consumption on the white blood cell count (WBC). The X-axis represents changes in alcohol consumption and the Y-axis represents changes in the WBC from the same persons in 2013 and 2018. The red dotted line represents approximate straight line.


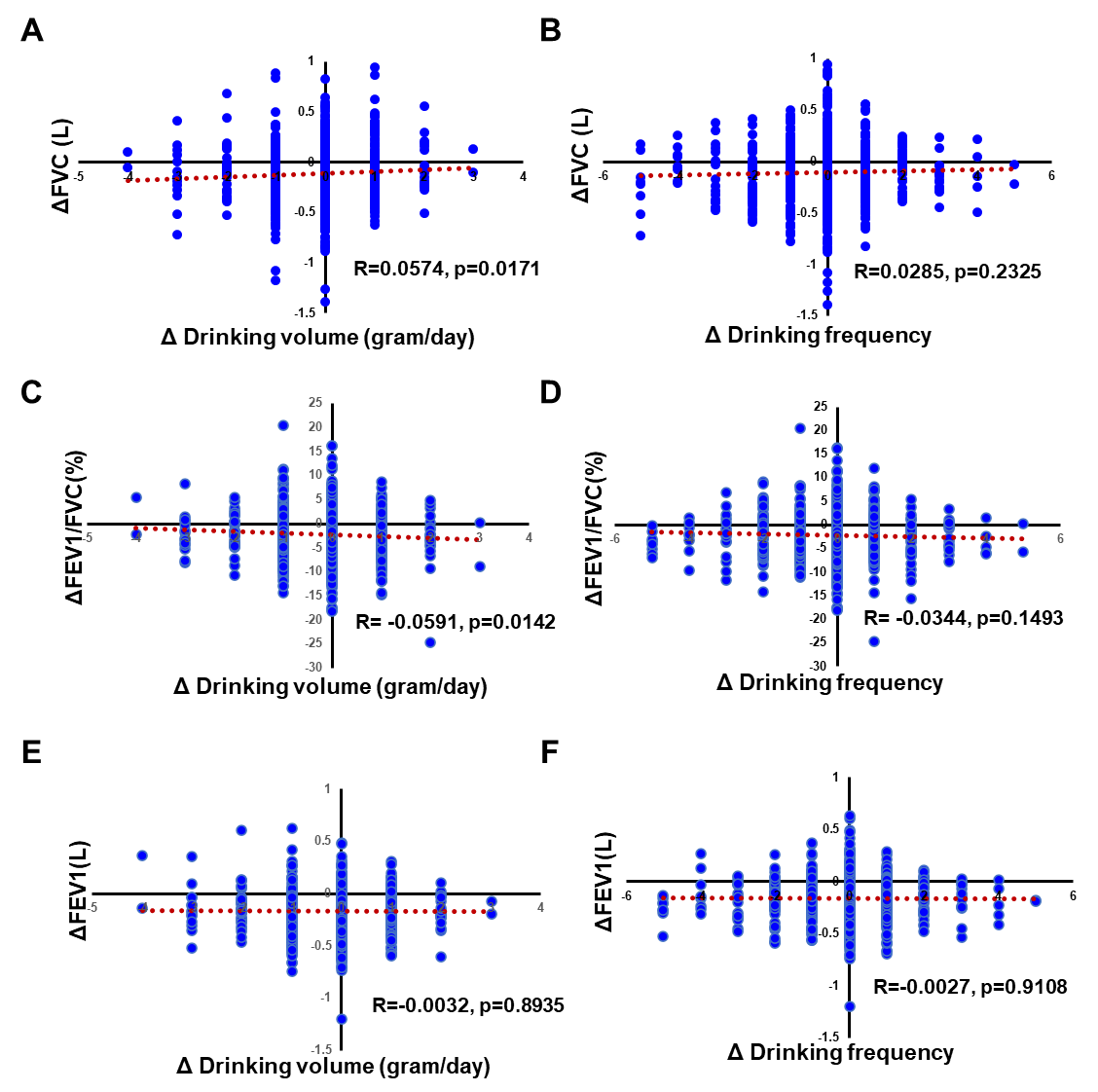


**Supplementary Fig. S4.** **Increases in drinking volume per day, but not drinking frequency, protect subjects against time-related physiological deterioration of FVC. A**, Time course of the effect of changes in drinking volume per day on time-related deterioration of FVC. The X axis represents the change in the category scoring of drinking volume per day and the Y axis represents the change in FVC from the same persons in 2013 and 2018. In this and the following subplots, the red dotted line represents an approximate straight line. **B**, Time course analysis of the effect of changes in drinking frequency on time-related deterioration of FVC. The X axis represents the change in the category scoring of drinking frequency and the Y axis represents the change in FVC from the same persons in 2013 and 2018. **C**, Time course analysis of the effect of changes in drinking volume per day on time-related deterioration of the FEV1/FVC ratio. The X axis represents the change in the category scoring of drinking volume per day and Y axis represents the change in the FEV1/FVC ratio from the same persons in 2013 and 2018. **D**, Time course analysis of the effect of changes in drinking frequency on time-related deterioration of FEV1/FVC ratio. The X axis represents changes in the category scoring of drinking frequency and the Y axis represents the changes in the FEV1/FVC ratio from the same persons in 2013 and 2018. **E**, Time course analysis of the effect of changes in drinking volume per day on time-related deterioration of FEV1. The X axis represents the change in the category scoring of drinking volume per day and the Y axis represents the change in FEV1 from the same persons in 2013 and 2018. **F**,Time course analysis of the effect of changes in the category scoring of drinking frequency on time-related deterioration of FEV1. The X axis represents the change in drinking frequency and Y axis represents the change in FEV1 from the same persons in 2013 and 2018.
