# Supplementary material for: Unbiased, comprehensive analysis of Japanese health checkup data reveals a protective effect of light to moderate alcohol consumption on lung function
Source: Sci Rep. 2021 Aug 5;11:15954. doi: 10.1038/s41598-021-95515-4 (PMC8342527; doi:10.1038/s41598-021-95515-4)
Supplement: Supplementary file 2 — Supplementary Tables. [file 41598_2021_95515_MOESM2_ESM.docx]

**Supplementary Table S1.** **Numbers and percentages of subjects that changed drinking habits from 2013 to 2018, segregated by sex.** Numbers and percentages of subjects for each degree of change in total alcohol consumption (gram per week) from 2018 to 2013 are segregated by sex. The degree of change in alcohol consumption of each individual was determined as the difference between the category scoring of total alcohol consumption in 2018 compared with that in 2013.

|  | Degree of change (Total alcohol consumption(gram/week)) | | | | | | | |
| --- | --- | --- | --- | --- | --- | --- | --- | --- |
|  | -350~-301 | -300~-251 | -250~-201 | -200~-151 | -150~-101 | -100~-51 | -50~-1 | 0 |
| Male,%(n) | 0.53(6) | 0.62(7) | 0.53(6) | 1.6(18) | 4.6(52) | 8.1(92) | 11.9(134) | 49.6(560) |
| Female,%(n) | 0.51(3) | 0.34(2) | 0.17(1) | 0.34(2) | 1.7(10) | 2.9(17) | 15.2(90) | 60.3(358) |
| Total %, (n) | 0.5(9) | 0.5(9) | 0.4(7) | 1.2(20) | 3.6(62) | 6.3(109) | 13.0(224) | 53.3(918) |
|  | Degree of change (Total alcohol consumption(gram/week)) | | | | | | | |
|  | 1~50 | 51~100 | 101~150 | 151~200 | 201~250 | 251~300 | Total | |
| Male,%(n) | 8.4(95) | 6.6(74) | 4.4(50) | 2.3(26) | 0.27(3) | 0.53(6) | 100(1129) | |
| Female,%(n) | 11.8(70) | 4.7(28) | 1.5(9) | 0.51(3) | 0(0) | 0.17(1) | 100(594) | |
| Total %, (n) | 9.6(165) | 5.9(102) | 3.4(59) | 1.7(29) | 0.2(3) | 0.4(7) | 100(1723) | |

**Supplementary Table S2.** **Numbers and percentages of subjects categorized by drinking habits and segregated by sex.** Subjects received health checkup in 2018 at Keio University Hospital and were categorized by drinking habits (volume consumed per day and frequency) and total alcohol consumption.

|  | | Male | Female |
| --- | --- | --- | --- |
| Drinking volume per day (gram) | None | 16.7% (617/3696) | 41.6% (974/2340) |
|  | <20 | 21.0% (777/3696) | 33.1% (774/2340) |
|  | 20-40 | 29.4% (1088/3696) | 17.7% (415/2340) |
|  | 40-60 | 21.4% (791/3696) | 5.5% (128/2340) |
|  | >60 | 11.1% (412/3696) | 1.6% (38/2340) |
|  | Data missing | 0.3% (11/3696) | 0.5% (11/2340) |
| Drinking frequency | None | 16.7% (619/3696) | 41.8% (977/2340) |
|  | A few times per month | 12.3% (455/3696) | 18.3% (429/2340) |
|  | Once –twice/Week | 13.4% (496/3696) | 13.3% (310/2340) |
|  | 3-4 times/ Week | 15.9% (588/3696) | 10.8% (252/2340) |
|  | 5-6 times/week | 18.1% (669/3696) | 7.4% (172/2340) |
|  | Everyday | 23.5% (867/3696) | 8.5% (199/2340) |
|  | Data missing | 0.1% (2/3696) | 0.0% (1/2340) |
| Total alcohol consumption | Never | 16.7% (619/3696) | 41.8% (977/2340) |
|  | Light | 24.4% (901/3696) | 34.9% (817/2340) |
|  | Moderate | 45.8% (1692/3696) | 20.6% (483/2340) |
|  | Heavy | 12.8% (473/3696) | 2.2% (51/2340) |
|  | Data missing | 0.3% (11/3696) | 0.5% (12/2340) |

**Supplementary Table S3.** **Characteristics of smoking habits in the study.**

**A**. **Smoking and drinking habits of patients who received a health checkup at Keio University Hospital in 2018.**

|  | | Smoking habit | | | | |  |
| --- | --- | --- | --- | --- | --- | --- | --- |
|  |  | Never | Previous | Current | Data missing | Total | |
| Drinking habit | Non-drinker | 1060 | 425 | 98 | 8 | 1591 | |
|  | Drinker | 1928 | 1932 | 542 | 41 | 4443 | |
|  | Data missing | 1 | 1 | 0 | 0 | 2 | |
|  | Total | 2989 | 2358 | 640 | 49 | 6036 | |

**B**. **Numbers and percentages of subjects who changed tobacco consumption from 2013 to 2018.**

|  | Degree of change in the consumption of cigarettes per day from 2013 to 2018 | | | | | | | | | |
| --- | --- | --- | --- | --- | --- | --- | --- | --- | --- | --- |
|  | -20 | -15 | -10 | -5 | 0 | 5 | 10 | 15 | 20 | total |
| Ratio (n) | 2.52% (21) | 0.96% (8) | 4.1% (34) | 2.64% (22) | 86.2% (719) | 1.44% (12) | 1.32% (11) | 0.12% (1) | 0.72% (6) | 100% (834) |

**Supplementary Table S4. An independent positive correlation between drinking habits and skeletal muscle mass/ strength** **calculated using three statistical algorithms.** Correlation values between drinking habits and skeletal muscle mass/ grip calculated by three statistical algorithms (ANOVA, CORR, and PCIT) using data from subjects who received a health checkup at Keio University Hospital in 2018 (n=6036).

|  | | Muscle mass | Grip |
| --- | --- | --- | --- |
| ANOVA | Drinking volume per day | 0.73 | 0.70 |
|  | Drinking frequency | 0.66 | 0.63 |
|  | Total alcohol consumption | 0.69 | 0.64 |
| CORR | Drinking volume per day | 0.47 | 0.39 |
|  | Drinking frequency | 0.34 | 0.27 |
|  | Total alcohol consumption | 0.39 | 0.30 |
| PCIT | Drinking volume per day | Significant | Significant |
|  | Drinking frequency | Significant | Significant |
|  | Total alcohol consumption | Significant | Significant |

**Supplementary Table S5. Items included in the health checkup, including** **the lifestyle-related questionnaire and clinical data**.

| **Clinical data** | |
| --- | --- |
| Age, Sex | |
| Body measurements | Height, body weight, BMI, abdominal circumference, visceral fat area, visceral fat / cutaneous fat ratio |
| Blood pressure, heart rate, eyesight, hearing | |
| Eyesight, hearing |  |
| Spirometry | FVC, FEV1, %FVC, %FEV1, FEV%, V50, V25, peak flow |
| Blood biochemical tests | WBC, RBC, PLT, Hb, Hct, MCV, MCH, MCHC, neutrophil(rods, segments), eosinophil, basophil, monocytes, lymphocytes, TP, Alb, GOT, GPT, LDH, ALP, gGTP, ChE, Amy, T-bil, D-bil, CPK, TG, TC, HDLc, LDLc, LPa, apoA, apoB, atherosclerosis index, FPG, HbA1c, insulin, HOMA-R, HOMA-b, UA, Cre, BUN, eGFR, Na, Ca, Cl, K, IP, CRP, TP-Ab, HBs-Ab, HBs-Ae, RF, HCV-Ab, Fe, TIBC,UIBC, CA125, PSA, adiponectin, PAI-1, cystatin C, BNP, intact PTH, osteocalcin, TSH, free T3, free T4 |
| Urinalysis | TP, gravity, pH, urobilinogen, ketone, nitrite, occult blood, RBC, WBC, bacteria, microalbumin, creatinine |
| FOB, Chest CT, Abodminal US, PWV,ABI, Bonemineral density, Skeletal muscle mass, grip | |
| **Questionnaires** | |
| Past medical history, Family medical history | Hypertension, DM, dyslipidemia, brain stroke, heart stroke, chronic renal failure, peripheral arterial disease,anemia, respiratory disease, GI tract disease, liver / gallbladder / pancreas disease,thyroid disease, hyperulicemia, rheumatic disease, osteoporosis,mortor disorder, psychiatric disorder, eye disease, ear / nose / throat disease, skin disease, urological disease, prostate disease, gynecological disease, cancer |
| Smoking habit | Are you a current / ex- / never- smoker? How many cigarettes per day? How long have you smoked? |
| Drinking habit | How much volume do you drink?(Never,<20g/day,20-40g/day,40-60g/day,≥ 60g/day), How often do you drink?(Never,few times per month,1-2days per week,3-4days per week,5-6 days per week,everyday) |
| Exercise habit | Do you exercise ≥ 30 minutes(yes or no) ≥twice per week?(yes or no) Is your walking speed fast?(slow, normal,fast) Do you walk daily ≥1 hour or do equivalent exercise?(yes or no) |
| Eating habit | Is your eating speed fast? (slow, normal,fast)Do you skip breakfast ≥3 times per week?(yes or no) Do you eat a snack after dinner ≥ 3 times per week?(yes or no) Do you eat dinner late at night ≥ 3 times per week?(yes or no) |
| Weight gain | Have you gained weight ≥10kg per 20 years (decrease,no change,increase) Or ≥ 3 kg per year? (decrease,no change, increase) |
| Sleeping habit | Is your sleeping time short?(≤4 hours,5-6 hours,7-8 hours,9-10 hours, ≥11hours) |
| Motivation for health improvement | Do you desire health guidance? (do not plan to improve,plan to improve within one year, plan to improve within 6 months,already working on improvement (for <6 months),already working on improvement (for ≥ 6 months) |

**Supplementary Table S6. Total alcohol consumption (g/week) was calculated by multiplying the mean level of drinking volume category and drinking frequency category.**

**A. Total alcohol consumption calculation.**

| Total alcohol consumption  (alcohol g/week) | | Drinking volume (alcohol g/day) | | | | |
| --- | --- | --- | --- | --- | --- | --- |
|  |  | 0g/day(0) | 0g/d~20g/d(10) | 20g/d~40g/d(30) | 40~60g/d(50) | >60g/d(70) |
| Drinking frequency (day/week) | |  |  |  |  |  |
| Never | (0) | 0 | 0 | 0 | 0 | 0 |
| Few times per month | (0.5) | 0 | 5 | 15 | 25 | 35 |
| 1-2days per week | (1) | 0 | 10 | 30 | 50 | 70 |
| 3-4days per week | (3) | 0 | 30 | 90 | 150 | 210 |
| 5-6days per week | (5) | 0 | 50 | 150 | 250 | 350 |
| everyday | (7) | 0 | 70 | 210 | 350 | 490 |

**B. Drinking category by total alcohol consumption.**

| Drinking category | Total alcohol consumption (alcohol g/week) |
| --- | --- |
| Never | 0 |
| Light | 5,10,15,25,30,35 |
| Moderate | 50,70,90,150,210,250 |
| Heavy | 350,490 |
